# Life Balance – a mindfulness-based mental health promotion program: conceptualization, implementation, compliance and user satisfaction in a field setting

**DOI:** 10.1186/s12889-015-2100-z

**Published:** 2015-08-01

**Authors:** Lisa Lyssenko, Gerhard Müller, Nikolaus Kleindienst, Christian Schmahl, Mathias Berger, Georg Eifert, Alexander Kölle, Siegmar Nesch, Jutta Ommer-Hohl, Michael Wenner, Martin Bohus

**Affiliations:** Central Institute of Mental Health, Mannheim, Heidelberg University, Heidelberg, Germany; AOK Baden-Württemberg, Villingen-Schwenningen, Germany; Department of Psychiatry and Psychotherapy, University of Freiburg, Freiburg im Breisgau, Germany; Wenner Burnout Prävention, Freiburg, Germany; Faculty of Health, University of Antwerp, Antwerp, Belgium; College of Health & Behavioral Sciences, Chapman University, Orange, USA

**Keywords:** Primary prevention, Mindfulness, Mental health, Health promotion, Well-being, Psychological resilience

## Abstract

**Background:**

Mental health disorders account for a large percentage of the total burden of illness and constitute a major economic challenge in industrialized countries. Several prevention programs targeted at high-risk or sub-clinical populations have been shown to decrease risk, to increase quality of life, and to be cost-efficient. However, there is a paucity of primary preventive programs aimed at the general adult population. “Life Balance” is a program that employs strategies borrowed from well-established psychotherapeutic approaches, and has been made available to the public in one federal German state by a large health care insurance company. The data presented here are the preliminary findings of an ongoing field trial examining the outcomes of the Life Balance program with regard to emotional distress, life satisfaction, resilience, and public health costs, using a matched control group design.

**Methods:**

Life Balance courses are held at local health-care centers, in groups of 12 to 15 which are led by laypeople who have been trained on the course materials. Participants receive instruction on mindfulness and metacognitive awareness, and are assigned exercises to practice at home. Over an 8-month period in 2013–2014, all individuals who signed up for the program were invited at the time of enrollment to take part in a study involving the provision of psychometric data and of feedback on the course. A control group of subjects was invited to complete the questionnaires on psychometric data but did not receive any intervention.

**Results:**

Of 4,898 adults who attended Life Balance courses over the specified period, 1,813 (37.0 %) provided evaluable study data. The average age of study participants was 49.5 years, and 83 % were female. At baseline, participants’ self-reported symptoms of depression and anxiety, life satisfaction, and resilience were significantly higher than those seen in the general German population. Overall, evaluations of the course were positive, and 83 % of participants attended at least at 6 of the 7 sessions. Some sociodemographic correlations were noted: men carried out the assigned exercises less often than did women, and younger participants practiced mindfulness less frequently than did older ones. However, satisfaction and compliance with the program were similar across all sociodemographic categories.

**Conclusions:**

While the Life Balance program is publicized as a primary prevention course that is not directed at a patient population, the data indicate that it was utilized by people with a significant mental health burden, and that the concept can be generalized to a broad population. As data from the control group are not yet available, conclusions about effectiveness cannot yet be drawn.

**Trial registration:**

German Clinical Trials Registration ID: DRKS00006216

## Background

Recent data from the World Health Organization (WHO) reveal that mental disorders account for 12.3 % of all disability-adjusted life years (DALYs) in the Americas and 10.9 % of DALYs in Europe [1]. These figures represent an enormous burden for individuals and their families, with 38.2 % of the European population – 164.8 million people – being affected by at least one mental disorder per year [2]. In Germany, mental disorders were the second highest cause of absenteeism due to illness in 2012, and the second highest cause of early retirement, accounting for 42 % [3]. In 2010, the total European costs for mental disorders were estimated at €418 billion, with 34.70 % due to direct health care costs, 12.11 % to direct non-medical costs, and 53.19 % to indirect costs [4].

The burden is even higher when subthreshold mental disorders, which are highly prevalent and pose a high risk for serious mental disorders, are taken into account [5]. Subthreshold mental disorders are associated with a decrease in health-related quality of life, increased use of health services, and productivity losses at the workplace due to ‘presenteeism’ — attending work while sick — which are estimated to be around 7–15 times more costly than the losses caused by absenteeism [6]. Even the absence of psychological well-being has been shown to increase the risk for mental disorders [7], which underscores the WHO’s claim that the promotion of well-being is as important as the reduction of mental illnesses [8].

Apart from treatment programs, effective prevention programs would help reduce the enormous burden of mental disorders. Research on prevention programs that are selective (aimed at high-risk groups) or indicated (aimed at persons with subclinical symptoms) has shown promising findings. However, these types of programs have some restrictions: they have limited accessibility; they carry an implication of labeling (and in the worst case, stigmatization); and they require screening of potential participants. In the last few decades, universal preventive programs (i.e., ones not targeted at patient populations) have been developed for children and adolescents [9]. However, little investigation has been done on the effectiveness of these programs for adults; and existing programs have a rather small sphere of influence, being available only in limited settings such as companies, universities, and the military [e.g. 10–16].

Two major challenges may be contributing to the relative paucity of universal primary mental health prevention programs for adults. First, assessment of effectiveness is hampered by a multitude of moderating variables, including the relatively low (for research purposes) incidence rates of mental disorders, and potential floor effects of outcome measures. To achieve adequate statistical power, a large number of subjects have to be included in evaluation studies, resulting in very costly and complex study designs [17]. Second, the systematic implementation of newly developed psychosocial treatments in naturalistic settings is scarce in all domains of mental health [18]. This is especially true in preventive mental health care, where resources, funding, and continued support are often rather low [19].

Accordingly, there is a pressing need for primary mental health programs to receive either more government funding or sponsorship from non-profit organizations or large health care insurance companies. In 2013, the German insurance company AOK Baden-Württemberg planned a region-wide health campaign with the aim of providing information on how to improve and consolidate balance in everyday life and work. To meet this goal, we developed a universal prevention program, based on current scientific knowledge, which should be appealing, motivating, and enjoyable for participants, easy to understand without the need for higher education, could be made available to the general public, and – for dissemination purposes – could be taught by psychological and medical laypeople rather than professionals. The goal was not to target specific or individual risk factors, but rather to promote protective factors for mental health in general and to enhance participants’ level of resilience. The scientific underpinnings of this program, titled “Life Balance”, are described below.

### Resilience and protective factors in mental health

Resilience is described by Rutten et al. as “a dynamic and adaptive process that subserves maintaining, or swiftly regaining, homeostasis in conditions of stress” [20; p.4]. This concept, along with the positive psychology movement, initiated a wealth of research on the protective nature of cognitive constructs and psychosocial factors. Although there is an ongoing debate whether fostering protective factors broadly prevents mental illness, there is considerable evidence for the protective value of a strong sense of coherence (the enduring tendency to perceive one’s environment as comprehensible, manageable, and meaningful [e.g. 21], high self-efficacy (the subjective belief in one’s ability to cope with challenging situations [e.g. 22], and the ability to build and maintain social support networks [e.g. 23]. Recent studies have added evidence for the protective value of self-compassion [e.g. 24], for being able to experience and to cultivate positive emotions [e.g. 25], and for experiencing purpose in life [e.g. 26].

Researchers in the field of resilience and protective factors have tried to show the primacy of putatively globally protective factors over maladaptive strategies. However, even the “classical” constructs have been shown to not be globally adaptive; for example, too much social support can pose a threat to self-esteem [e.g. 27]. The importance of situational flexibility in cognitive appraisal, emotion regulation, and coping strategies has therefore been increasingly highlighted in resilience research [28]. Bonanno and Burton [29] suggest sensitivity to context, availability of a diverse repertoire of regulatory strategies, and responsiveness to feedback to be prerequisites of resilience. Sensitivity and responsiveness require openness to reality and meta-cognitive as well as meta-emotional skills, as described in mindfulness practice and acceptance interventions [30, 31].

The practice of mindfulness, defined by Kabat-Zinn as “‘paying attention in a particular way: on purpose, in the present moment, and nonjudgmentally” [32; p.145], has been shown to be associated with increased subjective well-being and improved emotional as well as behavioral regulation [33]. A recent meta-analysis on mindfulness-based stress reduction for healthy adults found large effects on stress and moderate effects on anxiety, depression, distress, and quality of life [34].

Life Balance uses strategies derived from three therapeutic approaches. The psychological flexibility model that underlies Acceptance and Commitment Therapy (ACT; [35, 36]) offers an evidence-based concept that has already shown promising results in both indicated prevention programs and universal prevention programs [10, 37]. However, with respect to the literature on resilience, ACT targets only some protective factors. Therefore, we decided to additionally integrate some well-established strategies of two other mindfulness-based therapeutic approaches: Dialectical Behavioral Therapy (DBT; [38]), to enhance emotion regulation, social support, and communication; and Compassion Focused Therapy (CFT; [39]), to foster a self-compassionate stance.

### Program description

The Life Balance program comprises seven modules, each 1.5 hours long. Table 1 shows the focus of each module and identifies which of the therapeutic schools (ACT, DBT, or CFT) its interventions are derived from. The first six modules are held weekly, and the final module takes place four to six weeks after the sixth one, as a follow-up. The basic principles of mindfulness and metacognitive awareness are addressed in all the modules, to enable a sustainable learning process. In between the sessions, participants are given homework (called balance exercises), in order to enhance the implementation of the course content in everyday situations; and are encouraged to perform regular mindfulness exercises. In didactic terms, apart from conveying knowledge, the course adopts an experiential approach.

**Table 1:** Overview of life balance program

Module	Interventions	Aimed at enhancing the following protective factors
1: Mindfulness	Mindfulness exercises^ab^	Metacognitive awareness
2: Compassionate inner coach	Psychoeducation, self-compassion-exercises^c^	Self-compassion and -worth
3: Values	Evaluation of one’s own values^b^	Sense of coherence, purpose in life
4: Social networks and validating communication	Social network analysis and communication training^a^	Social support and social communication skills
5: The art of change	Problem-solving^a^	Self-efficacy, flexible coping
6: Dealing with obstacles	Defusion,^b^ acceptance^a^	Emotion regulation, cognitive flexibility
7: Reflection and consolidation	Behavior analysis^a^	Self-efficacy

^a^DBT

^b^ACT

^c^CFT

In Module 1, the fundamental principles of the program are explained, and participants acquire the basic mindfulness skills of openness to experience and acceptance of both reality and their own mental and physical state in an intentional and non-judgmental way. In Module 2, a metacognitive point of view is used to differentiate between exaggerated self-critical thoughts and features of the actual situation, and to build a self-compassionate self-image. Module 3 targets enhancing awareness of individual values as a basis for formulating specific, cross-situation life goals following the ‘theory of universal values’ [40]. In Module 4, size, quality, stability, and diversity of individual social networks are analyzed, and validating communication skills are taught in role plays to reinforce the stability of social relationships. In Module 5, strategies are taught for increasing individuals’ problem-solving abilities which can be used both to cope with difficult situations and to implement behavioral changes in daily life. Module 6 deals with obstacles in the process of behavior change and/or living according to one’s values. Contextual obstacles are discussed, but the focus lies on dealing with dysfunctional thoughts and accepting difficult emotions. Participants commit themselves to practicing the newly acquired skills in individual behavior change projects (called “Balance Projects”), which are evaluated in Module 7.

## Methods

### Program development and implementation

The costs of developing and implementing the Life Balance program were covered by the health care insurance company AOK Baden-Württemberg. The program was first tested in two pilot courses with qualitative formative evaluation, and was then tested for feasibility and acceptance with 1,272 of the sponsor’s employees. Since October 2013, it has been offered in the federal state of Baden-Württemberg, publicized by the sponsor via mailings, public presentations, flyers, and radio ads. The advertisements are designed to carry a positive message, avoiding the term “mental health”. The courses take place in local health centers, with enrollment of 12 to 15 participants, and are led by over 200 employees of AOK Baden-Württemberg who mainly hold degrees in sports or nutrition and have experience in conducting prevention group programs. The presenters receive three days of training from the program developers, have access thereafter to an online supervision tool, and attend a one-day supervision group during the program implementation. Courses are presented in accordance with a structured manual, standard presentation slides, and handouts for participants. As optional supplementary materials, a self-help book [41] and a CD demonstrating mindfulness exercises [42] are available from bookshops.

### Evaluation study

The data presented in this article are part of a large ongoing field evaluation of the program that aims to examine the outcomes in terms of emotional distress, life satisfaction, resilience, and public health costs, using a matched control group design. Subjects in the control group, who completed the questionnaires on psychometric data without having taken part in the Life Balance program, were drawn from the pool of policy-holders at AOK Baden-Württemberg and were matched with the program participants using propensity score matching. Here, we report on baseline characteristics of the study sample, as well as the participants’ compliance and satisfaction with the program. Since the collection of outcome data will not be completed until the autumn of 2015, data on the control group, including the matching process and results concerning effectiveness, will be reported in a subsequent publication.

This study was registered in the German Clinical Trials Registration database (ID DRKS00006216), and approval was obtained from the ethics review committee of the University of Heidelberg (approval number: 2013620NMA).

### Participants

Study participants were recruited from all those who registered in a Life Balance course between November 2013 and June 2014. Inclusion criteria were age ≥18 years, sufficient German language skills, and capacity to give informed consent. It was explained that agreeing to take part in the study was optional and was not a precondition for being in the course; thus, the sample was completely self-selected.

Demographic data were collected from everyone who enrolled in a Life Balance course, while psychometric data and feedback on the course were collected only from the subset who agreed to be in the study.

### Data collection and measures

Data collection was carried out via a battery of self-administered psychological questionnaires. Measurements were conducted prior to participation in the course (t0), immediately after completing the course (t1 = t0 + 10 weeks), 3 months after completion (t2 = t0 + 22 weeks), and 12 months after enrolment (t3 = t0 + 12 months). Only data from the t0 and t1 time points are presented here; findings obtained at t2 and t3 will be provided in a future publication.

#### Baseline measures

The *Hospital Anxiety and Depression Scale* (HADS; [43]) measures symptoms of depression (7 items) and anxiety disorders (7 items) over the past week, using two subscales. Items are rated on a 4-point scale. The HADS has good psychometric properties, with a reported internal consistency (Cronbach’s α) greater than .80, a high level of acceptance in non-clinical samples, and international use in screening for mental disorders [44]. Sensitivity and specificity of the HADS in the clinical diagnosis of depressive disorders are .82 and .74, respectively [45].

The *Resilience Scale*, 11-item short version (RS-11 [46], German version [47]) measures resilience “as the ability to use internal and external resources successfully to cope with developmental tasks” [47, p. 21]. Items are rated on a 7-point scale ranging from (1) “strongly disagree” to (7) “strongly agree”. The item scores are summed, with higher scores indicating higher resilience. The scale has good psychometric properties, with a reported internal consistency of α = .81.

The *Satisfaction with Life Scale* (SWLS; [48]) is a one-dimensional scale (5 items) that rates life satisfaction as a global, personal assessment of one’s own life [49]. Respondents indicate how much they agree with each item on a 7-point scale ranging from (1) “strongly disagree” to (7) “strongly agree”. Item scores are summed, with higher scores indicating higher satisfaction. The SWLS has good psychometric properties, with a reported internal consistency of α = .92 [48].

#### Evaluative measures

Participants’ feedback on the course was assessed through program-specific questions using a 5-point visual analogue scale at the t1 (appraisal), t2 (compliance), and t3 (compliance) time points. Only data from t1 are provided here.

Specific and non-specific health costs will be drawn from the insurance company’s stock data, and will be provided in a future publication.

### Data analysis

Descriptive statistics were used to analyze the sociodemographic data and evaluative measures. To compare the sociodemographic characteristics of the subset of participants who took part in the research study against the complete sample of course participants, (as well as research participants responding to the second measurement compared to those who did not), t-tests were used for continuous variables, and chi-square tests for dichotomous variables. Baseline psychometric data were compared to German norm values for the respective questionnaires using *t*-tests.

## Results

### Study population

**Fig. 1:**
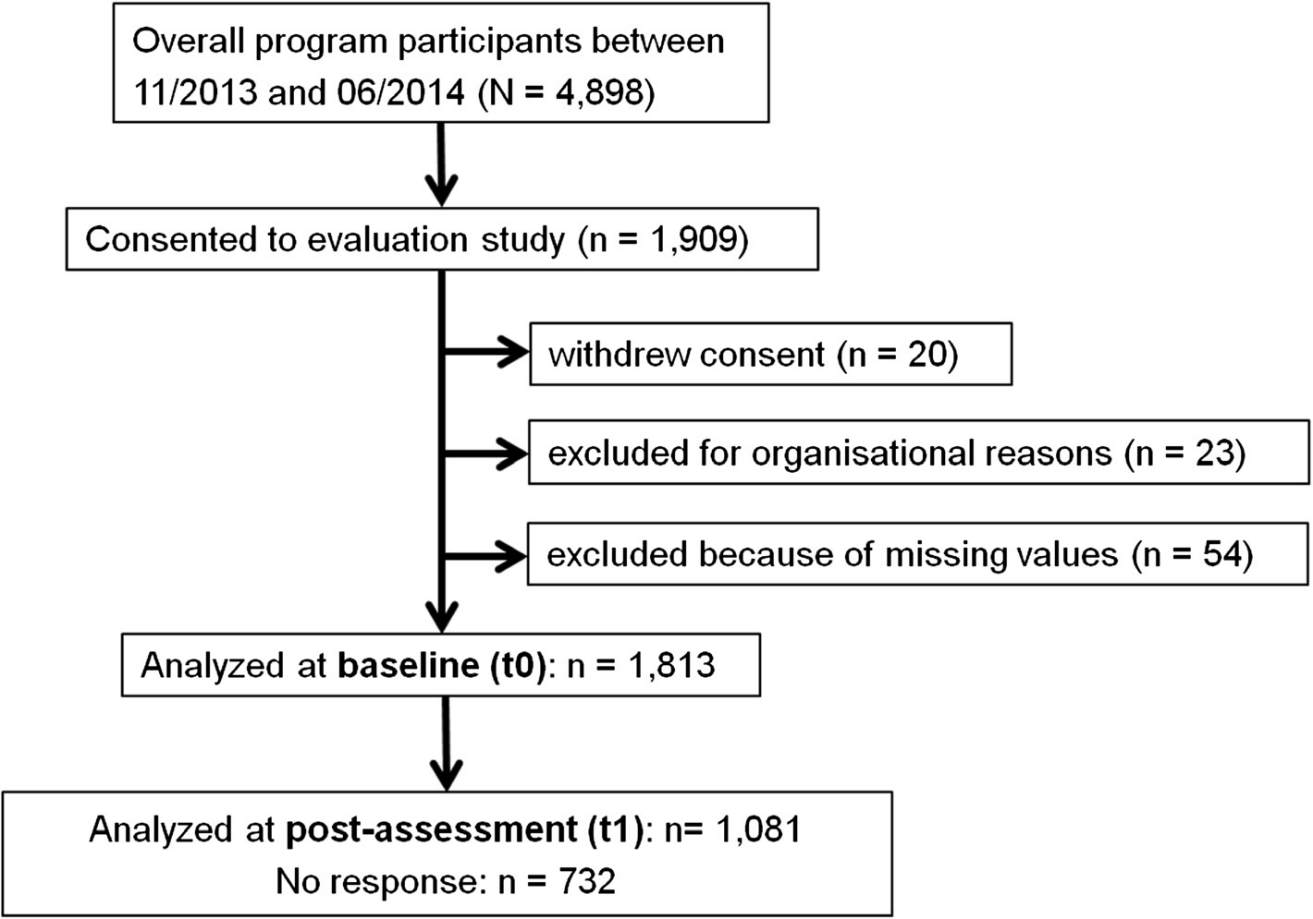
Flow chart of participants

The participant flow is shown in Fig. 1. Of the 4,898 persons who enrolled in a Life Balance course between November 2013 and June 2014, 173 did not receive an invitation to participate in the study due to organizational delays. A total of 1,910 agreed to take part, of whom 20 subsequently withdrew, while 77 others had to be excluded from the database either because their data could not be clearly attributed due to questionnaires having been sent out twice in error (*n* = 23) or because more than 50 % of items in the baseline questionnaires were missing (*n* = 54). In the end, the data of 1,813 individuals, representing 37.0 % of the total who enrolled in the program, were included at baseline. At the second measurement time point (t1 = t0 + 10 weeks), 1,074 participants (59 % of the baseline sample) provided data.

Baseline sociodemographic data are shown in Table 2. Of the 1,813 study participants, 1,506 (83 %) were female and 307 (17 %) were male. Average age was 49.5 years (SD = 11.4; range = 18–87). With regard to family status, 59 % were married, 68 % lived with a spouse or partner, and 51 % had children. With regard to highest level of education, 22 % had 9 years of schooling (a basic School Leaving Certificate), 44 % had 10 years, and 32 % had 12 years or more (at least a High School Leaving Certificate). Age and educational level did not differ significantly between the subset of program participants who took part in the study and the total sample of participants. A difference was however seen for gender: 32 % of all male and 38 % of all female course participants agreed to take part in the evaluation study (Χ^2^(1) = 11.187, *p* = .001).

**Table 2:** Baseline sociodemographic characteristics

		Female	Male	Total
		(*N* = 1,506)	(*N* = 307)	(*N* = 1,813)
Age	M	49.18	50.97	49.48
	SD	11.45	10.67	11.34
	Range	18–87	28–82	18–87
Age groups	18–30 years	6.0 %	2.0 %	5.3 %
	31–40 years	15.8 %	11.7 %	15.1 %
	41–50 years	31.1 %	36.8 %	32.0 %
	51–60 years	33.5 %	33.9 %	33.5 %
	61–70 years	9.8 %	10.7 %	10.0 %
	>71 years	3.9 %	4.9 %	4.0 %
Education	No school leaving certificate	0.6 %	0.3 %	0.6 %
	9 yrs. of education	21.6 %	28.3 %	22.7 %
	10 yrs. of education	46.9 %	32.2 %	44.4 %
	≥ 12 yrs. of education	30.9 %	39.1 %	32.3 %
Marital status	Married	57.6 %	64.6 %	58.9 %
	Single	19.1 %	22.3 %	19.7 %
	Divorced	16.3 %	11.1 %	15.5 %
	Widowed	5.4 %	0.7 %	4.6 %
	Common law	1.3 %	1.3 %	1.3 %
Living with spouse or partner	Yes	67.8 %	73.9 %	68.8 %
	No	30.8 %	26.1 %	30.0 %

The subset of participants who provided data at the second time point (t1) did not differ significantly at baseline from the overall study sample on any of the sociodemographic variables or psychometric measures.

### Psychometric data at baseline

Baseline psychometric data are shown in Table 3. The participants differed significantly from norm values for the general German population (representative population surveys [47, 48, 50]) in all primary outcome measures: more symptoms of depression and anxiety (women: t(3985) = 16.72, *p* < .0001; men: t(2234) = 14.80, *p* < .0001; [50]), less life satisfaction (women: t(2820) = 12.97, *p* < .0001; men: t(1,508) = 8.52, *p* < .0001; [48]), and lower resilience scores (t(3,814) = 11.01, *p* < .0001; [47]).

**Table 3:** Baseline data on the primary outcome measures in comparison to German norm values

		Evaluation of life balance			Norm values of German population [50]			Unpaired *t*-tests	
		m	SD	*n*	M	SD	*n*	t	*p*
HADS	Female	15.75	6.98	1,506	9.7	6.9	2,481	16.72	< .0001
	Male	15.37	7.29	307	9.2	6.7	1,929	14.80	< .0001
	Total	15.69	7.03	1,813	9.45	6.8	4,410	32.57	< .0001
					Norm values [48]				
		m	SD	*n*	M	SD	*n*	t	*p*
SWLS	Female	21.57	6.45	1506	24.67	6.20	1,315	12.97	< .0001
	Male	21.67	6.37	307	25.12	6.32	1,204	8.52	< .0001
	Total	21.59	6.44	1,813	24.88	6.26	2,519	21.98	< .0001
					Norm values [47]				
		m	SD	*n*	M	SD	*n*	t	*p*
RS	Female	54.29	11.45	1,506	n.a.	n.a.	1,070	n.a.	n.a.
	Male	53.02	11.40	307	n.a.	n.a.	934	n.a.	n.a.
	Total	54.07	11.45	1,813	58.03	10.76	2,003	11.01	< .0001

Using the criterion of a score ≥8 on the HADS-D scales, 57.9 % of female participants and 52.1 % of male participants had scores indicative of clinically relevant symptoms on at least one of the scales, compared to 33 % and 29 % of the general female and male populations, respectively [50]. With regard to severity of symptoms, 26.9 % of the sample had scores rated as mild (8–10), 34.1 % as moderate (11–15), and 5.5 % as severe (≥16) [51].

### Course appraisals

Table 4 presents participants’ appraisals of the course at the t1 time point. All questions used a 5-point visual analogue scale, with higher values indicating stronger agreement with the question. Responses were largely positive (scores of 4 or 5), with 76 % of participants stating that they were satisfied or very satisfied with the course, 81 % that they would recommend it to others, 77 % that its contents would help them in their daily lives, 66 % that the course had had a positive effect on their mental stability, and 61 % that it would enrich their lives. Appraisals of the course trainers with respect to commitment, teaching strategy, competence, and openness to questions were positive on average, with 79–89 % (depending on the question) of participants providing a rating of 4 or 5. There was no significant correlation between age, gender, and educational level of participants and their satisfaction with the course or the presenters.

**Table 4:** Participants’ appraisal of the course

	n	Mean value	SD
How satisfied were you with the Life Balance course as a whole?	1,114	4.09	.969
Would you recommend the Life Balance course to others?	1,112	4.28	1.016
Do you think that what you learned in the course will be useful in your daily life?	1,112	4.10	.942
Do you believe that what you learned in the course has a positive effect on your mental stability?	1,107	3.79	1.082
Do you believe that what you learned in the course will enrich your life?	1,110	3.95	1.009
Our course presenter was committed and motivated.	1,108	4.56	.798
Our course presenter taught well.	1,106	4.37	.931
Our course presenter was competent.	1,107	4.23	1.062
Our course presenter answered participants’ questions and responded to contributions.	1,109	4.45	.903

### Compliance

Course attendance was good, with 83 % of participants attending at least 6 of the 7 sessions. Completion of homework assignments varied according to the task: 89 % of participants reported that they had completed the value profile, 50 % that they had done the exercise to enhance metacognitive awareness of self-critical thoughts, 45 % that they had practiced mindfulness exercises more than twice a week during the course, and 97 % that they had done so at least once a week during the course. After the course was completed, the percentage saying that they were still doing this at least once a week dropped to 72 %. There were notable correlations with socio-demographic characteristics: Men carried out the homework tasks significantly less often than did women (Mann–Whitney-Test U(779,151) = 4,6832.5, *z* = −4.15, *p* < .000), and younger participants practiced mindfulness significantly less frequently than younger ones, both during and after the course (Pearson’s *r* = .105, *p* = .001 and *r* = .208, *r* < .0001, respectively).

## Discussion

This study evaluated the compliance and satisfaction of participants enrolled in a mindfulness-based course that was aimed at the prevention of mental health problems in adults. The findings reported, are a subset of the data being collected as part of a more extensive effectiveness trial which includes a control group.

Life Balance courses were designed as a universal primary preventive program; however, the self-selected study participants showed evidence of carrying a significant mental health burden, with psychological stress scores significantly above the norm for the German population. The sociodemographic data revealed a disproportionate utilization of the program by middle-aged women; the sample was 83 % female, with a mean age of 49.5 years. It is known from general population-based disease prevention programs that women, people over the age of 30, and people with a higher socioeconomic status or higher education are more likely to engage in preventative health behavior actions [52–55]. The high percentage of women participating in our study could be related to gender disparities in attitudes toward mental health and utilization of mental health services. There is evidence from a Canadian health survey that men may be more likely to avoid seeking help, especially for minor mental health concerns [56]. In a large European survey, more men stated that they would “feel uncomfortable talking about personal problems” and would “be embarrassed if friends knew about professional help” [57]. Both of these issues could be a concern when considering participation of males in a mental health prevention program.

As this trial is an evaluative field study in a naturalistic setting, research participation was voluntary and no prerequisite of participating in the prevention courses. About 40 % of all course participants agreed to take part in the evaluation study. One possible reason for this low rate could be a reluctance to participate in research being sponsored by an insurance company. In a review of barriers to participation in mental health research, Woodall et al. [58] identified concerns about confidentiality and suspicion or distrust of researchers as important factors. Although we explained to course participants that any data they provided would be kept completely confidential, it is likely that some had concerns about disclosure of their mental health status. A systematic analysis of selection effects in non-experimental evaluation studies is close to impossible, due to the large number of mostly unknown moderator variables [59, 60].

Following completion of the course, 59 % of the study sample returned the second set of questionnaires. The subset who responded had not differed significantly at baseline from the entire sample in terms of sociodemographic variables, psychological stress, life satisfaction, or resilience. Although the possibility of unknown moderator variables cannot be ruled out, this subset therefore does not seem to differ systematically from those who did not provide follow-up data. Methods for increasing the retention of research participants, such as offering incentives or contacting participants multiple times [see 58], were not used in our study.

Due to the organizational complexity of this multi-site study, it was not possible to monitor program attrition systematically. Therefore, it is not possible to link research attrition to program attrition. Informal counting suggests a dropout rate of about 20 % of all participants, which is within the range of drop-outs in prevention programs in sports and nutrition offered by the sponsoring insurance company. In the experience of the course instructors, the fact that the courses are offered free of charge to clients of the cooperating insurance company results in quite a few individuals signing up “just to see what it is”, without a strong commitment to take part. Reports on attrition in programs as well as in research studies vary substantially. A review of parent and child mental health programs found dropout rates ranging from 20 % to 80 % [59]; a meta-analysis of HIV prevention programs intended for at-risk groups calculated an average dropout rate of 25 % [60]; and a meta-analysis of mixed health behavior change interventions presents a mean attrition rate of 18 % in the intervention groups [61].

A study on the effectiveness of preventative interventions found that perceiving a program to be helpful and of high quality has an impact on its effect [62]. Participants’ satisfaction with the Life Balance courses was high, as were their commitment, motivation, and ratings of the course presenters’ teaching skills – the last being notable since the trainers were laypeople without professional training in psychology or medicine. Attendance rates and the performance of regular homework exercises in everyday life were high (with the exception of the exercise on self-critical thoughts), although we cannot rule out the possibility that participants who did not respond at the post-intervention measurement point had dropped out of the course or only attended it at irregular intervals. To our knowledge, there has been no comparable evaluation of a prevention program that has collected data on participants’ evaluation of course content and presentation in detail.

Some correlations with sociodemographic characteristics were seen: male participants practiced the balance exercises significantly less often than did women, and younger participants carried out fewer mindfulness exercises than did older ones. It may be that these sub-populations would be better served by using different imagery, motivational structure, and presentation of topics. These differences reflect a dilemma inherent in universally applicable prevention programs: they are likely to result in smaller effect sizes than those expected from indicated and selective programs, which are designed for specific target groups [63]. However, it is often not feasible to offer a variety of different programs, especially in rural areas. Apart from these differences, there was little correlation between the satisfaction and compliance ratings and any of the socio-demographic characteristics, which suggests that the concept of the Life Balance program could be applicable across different target populations.

### Limitations

Some limitations to the study must be noted. Systematic monitoring of the program implementation, as suggested in the literature [18], was not feasible for several reasons. First, the course instructors were employees of the sponsor, whose employee data protection policy did not allow us to conduct objective ratings of instructor adherence and performance ratings. Second, the courses took place in local health centers so there was no uniform data collection policy beyond participant registration, which made systematic assessment of implementation too complex for the means of this study. Therefore, it will not be possible to link implementation quality with outcome data, which may possibly result in an underestimation of effects that might have been seen in an optimal setting [19].

## Conclusion

The mindfulness-based prevention program “Life Balance” is based on research on resilience and protective factors for mental health, and uses evidence-based intervention strategies from psychotherapy research to strengthen those protective factors. Preliminary data from the implementation of this program in a field setting found that its contents are well accepted by participants from a wide range of socioeconomic backgrounds, attendance rates are high, and compliance with completion of assignments is good, all of which indicate that the program appears to be suitable for universal preventive purposes.

## Abbreviations

ACT: Acceptance and Commitment Therapy
CFT: Compassion Focused Therapy
DALY’s: disability-adjusted life years
DBT: Dialectical Behavioral Therapy
HADS: Hospital Anxiety and Depression Scale
RS: Resilience Scale
SD: Standard Deviation
SWLS: Satisfaction with Life Scale
